# Mutual stain conversion between Giemsa and Papanicolaou in cytological images using cycle generative adversarial network

**DOI:** 10.1016/j.heliyon.2021.e06331

**Published:** 2021-02-24

**Authors:** Atsushi Teramoto, Ayumi Yamada, Tetsuya Tsukamoto, Yuka Kiriyama, Eiko Sakurai, Kazuya Shiogama, Ayano Michiba, Kazuyoshi Imaizumi, Kuniaki Saito, Hiroshi Fujita

**Affiliations:** a Graduate School of Health Sciences, Fujita Health University, Aichi, Japan; b School of Medicine, Fujita Health University, Aichi, Japan; c Faculty of Medical Technology, School of Medical Sciences, Fujita Health University, Aichi, Japan; d Faculty of Engineering, Gifu University, Gifu, Japan

**Keywords:** Giemsa stain, Papanicolaou stain, Translation, Cycle-consistent generative adversarial network, Deep learning

## Abstract

**Objective:**

Papanicolaou and Giemsa stains used in cytology have different characteristics and complementary roles. In this study, we focused on cycle-consistent generative adversarial network (CycleGAN), which is an image translation technique using deep learning, and we conducted mutual stain conversion between Giemsa and Papanicolaou in cytological images using CycleGAN.

**Methods:**

A total of 191 Giemsa-stained images and 209 Papanicolaou-stained images were collected from 63 patients with lung cancer. From those images, 67 images from nine cases were used for testing and the remaining images were used for training. For data augmentation, the number of training images was increased by rotation and inversion, and the images were pipelined to CycleGAN to train the mutual conversion process involving Giemsa- and Papanicolaou-stained images. Three pathologists and three cytotechnologists performed visual evaluations of the authenticity of cell nuclei, cytoplasm, and cell layouts of the test images translated using CycleGAN.

**Results:**

As a result of converting Giemsa-stained images into Papanicolaou-stained images, the background red blood cell patterns present in Giemsa-stained images disappeared, and cell patterns that reproduced the shape and staining of the cell nuclei and cytoplasm peculiar to Papanicolaou staining were obtained. Regarding the reverse-translated results, nuclei became larger, and red blood cells that were not evident in Papanicolaou-stained images appeared. After visual evaluation, although actual images exhibited better results than converted images, the results were promising for various applications.

**Discussion:**

The stain translation technique investigated in this paper can complement specimens under conditions where only single stained specimens are available; it also has potential applications in the massive training of artificial intelligence systems for cell classification, and can also be used for training cytotechnologist and pathologists.

## 1 Introduction

Cytology, which allows the evaluation of the structure of nuclei, cytoplasmic properties, and cell layout taken from living organisms, plays an important role in pathological diagnosis. Among the staining methods employed in cytology, Papanicolaou and Giemsa stains are major staining techniques.

Papanicolaou staining is a general multichromatic staining method for cytology, which allows cells to be stained with three different dyes via wet fixation [1]. It is possible to observe cell aggregation and to make detailed observations of chromatin structure. However, this staining process is cumbersome and is often difficult to adapt to situations in which rapid specimen preparation is required.

In contrast, Giemsa staining is a method of staining cells with two types of blue dyes [2]. Specimens can be prepared in a short period of time with few staining steps. In addition, it has good fixation properties, which allows for the creation of specimens even when only a small number of cells can be collected. It is used for the rapid diagnosis and differentiation of benign and malignant cells. However, it is not possible to prepare specimens from liquid samples at the time of biopsy because of the dry method. In addition, compared to Papanicolaou staining, it is less proficient to delineate nuclear and cytoplasmic structures, making it difficult to differentiate the tissue type.

Because of the trade-off between the two staining methods, as described above, it is desirable to use both of these to make a diagnosis. However, it is often difficult to prepare both stains due to the limitations imposed by the collecting method of the cells.

Here, if the image of one stained specimen can be converted to another type of image, it can be used for diagnosis and other purposes. Therefore, we focused on a domain transformation technique, which is one of the deep learning techniques [3, 4, 5, 6, 7, 8, 9, 10, 11].

This is a method of transforming an image of one category into another type of image, and it has been proposed to transform the style of a photograph or painting, or to redraw an object in an image, into another type of object. In this study, we focused on cycle-consistent generative adversarial network (CycleGAN), a domain transformation technique [10]. Regarding medical applications, Jelmer et al. successfully converted magnetic resonance images into computed tomography (CT) images with CycleGAN [11]. If it is possible to convert Papanicolaou- and Giemsa-stained images into each other, it may be possible to generate alternative stained images, even when only one of them can be prepared, and this technology may be applied in clinical practice. To the best of our knowledge, no study has been reported till date on the conversion of Giemsa and Papanicolaou staining using deep learning.

The main contribution of this study is to propose a method for a mutual conversion of Papanicolaou- and Giemsa-stained images using CycleGAN, a domain transformation technique, and to discuss their applicability to diagnosis based on subjective evaluation by experts.

## 2 Materials and methods

### 2.1 Image dataset

**Figure 1:**
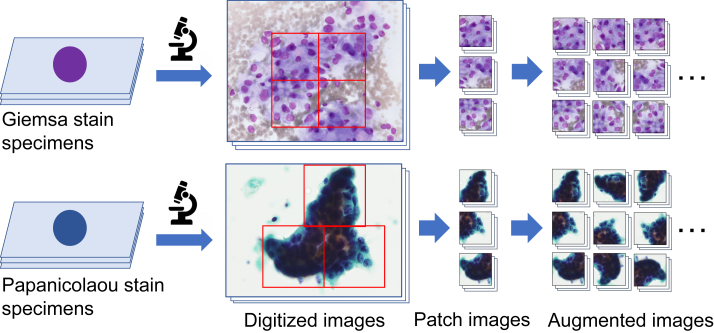
Image cropping and augmentation.

Lung cell samples from 63 patients with lung cancer were collected with interventional cytology using either bronchoscopy or CT-guided fine-needle aspiration cytology and comprised 43 cases of adenocarcinoma and 20 cases of squamous cell carcinoma according to combined histopathological and immunohistochemical diagnoses. The procedure of image preparation for this study is shown in Figure 1.

Regarding specimens for Papanicolaou staining, we applied liquid-based cytology using the BD SurePath™ liquid-based Pap test (Beckton Dickinson, Franklin Lakes, NJ, USA), with staining based on Papanicolaou's method. Using a digital still camera (DP70, Olympus, Tokyo, Japan) attached to a microscope (BX51, Olympus) with a × 40 objective lens, 142 images of adenocarcinoma, and 67 images of squamous cell carcinoma were collected in JPEG format. The initial matrix size of each JPEG image was 2040 × 1536 pixels. Subsequently, 768 × 768 pixels square images were generated via cropping, and these were further resized to 256 × 256 pixels.

Specimens were prepared using the Giemsa staining method using a brush-scraped specimen at the time of bronchoscopy. Using a digital still camera (Axiocam 506 color, Carl Zeiss, Jena, German) attached to a microscope (Axio Imager A1, Carl Zeiss) with a × 40 objective lens, 146 images of adenocarcinoma, and 45 images of squamous cell carcinoma were acquired in JPEG format. The initial matrix size of each JPEG image was 2752 × 2208 pixels. Subsequently, 706 × 706 pixels square images (same field of view as Papanicolaou images) were generated via cropping and were further resized to 256 × 256 pixels. The final image resolution input to CycleGAN was 3.2 μm per pixel.

From those images, 5 cases (45 images) of adenocarcinoma and 4 cases (22 images) of squamous cell carcinoma were used for testing and the remaining images were used for training.

Training of CycleGAN requires a large amount of data, as a small dataset may cause mode collapse [12]. To prevent overfitting, we augmented the training dataset using image processing. Microscopic images are direction invariant, and the sharpness of the target cell in each image varies according to the position of the focal plane of the microscope. Therefore, we performed data augmentation via rotating, flipping, smoothing, and sharpening of the original images [7].

This study was approved by an institutional review board (Fujita Health University), and patient consent was obtained under the condition that all data were anonymized (number HM16-155).

### 2.2 CycleGAN architecture

**Figure 2:**
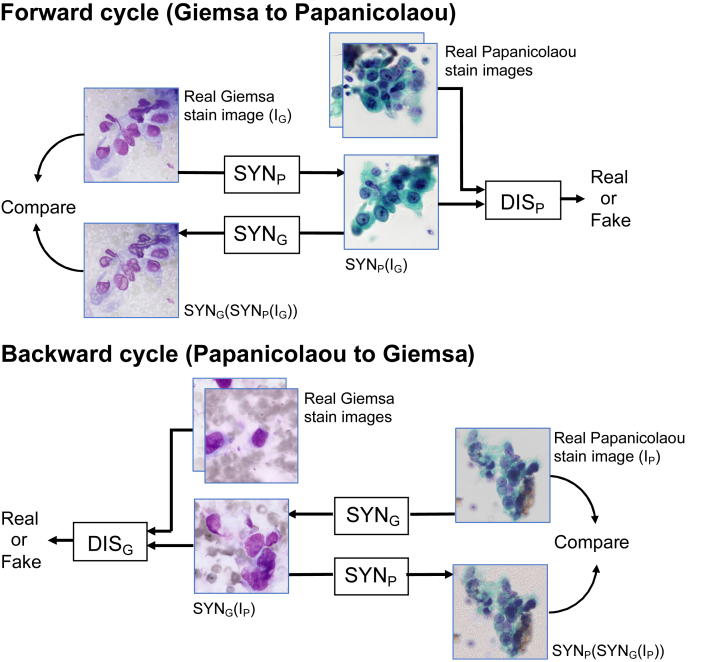
Cycle GAN architecture.

The architecture of CycleGAN used for image translation in this study is shown in Figure 2. The CycleGAN architecture consists of two cycles - a forward cycle and a backward cycle. In the forward cycle, a synthesis network, SYN_P,_ is trained to translate a given Giemsa-stained image I_G_ into a Papanicolaou-stained image; network SYN_G_ is trained to translate the resulting Papanicolaou-stained image back into a Giemsa-stained image that redraws the original Giemsa-stained image. DIS_P_ discriminates between real and synthesized Papanicolaou-stained images; it is trained to accurately classify real and synthesized Papanicolaou-stained images.

Similarly, in the backward cycle, SYN_G_ synthesizes Giemsa-stained images from given Papanicolaou-stained images; SYN_P_ reconstructs the Papanicolaou image from the synthesized Giemsa-stained image, and DIS_G_ discriminates between real and synthesized Giemsa-stained images.

To enforce bidirectional translation, reconstructed images of SYN_G_(SYN_P_(I_G_)) and SYN_P_(SYN_G_(I_P_)) were compared with real Giemsa-stained images and real Papanicolaou-stained images, respectively.

As for the implementation of CycleGAN for this study, we introduced Python code using TensorFlow by Xiaowei Hu [13]. As for architecture of SYN_G_ and SYN_P_, ResNet having nine residual blocks [14] was used, and Patch GAN [15] was used for DIS_G_ and DIS_P_. The procedure for determining the parameters of CycleGAN was as follows. After each epoch of training, the generated images were monitored to see whether there were any abnormalities such as pixel value inversion or artifacts. In most cases, these problems were resolved as the number of training epochs increased. However, if the output of abnormal images persisted even with further training, we changed the training coefficient and ran the training process again. As a result, we set the number of training epochs as 200, the learning rate as 0.0002, and beta as 0.5 for the Adam optimization algorithm [16]. A Python program developed using Keras and TensorFlow was executed on a computer equipped with AMD Ryzen 9 3900X as a CPU and NVIDIA TITAN RTX as a GPU. During the training of CycleGAN, the image quality of the generated images was checked, and the training process was carried out while confirming the stability of the generation.

### 2.3 Evaluation

Visual evaluation of the images converted by CycleGAN was performed. Among the image data set, nine cases in which both Giemsa and Papanicolaou stains were available were used for evaluation. These consisted of 33 Giemsa-stained images and 34 Papanicolaou-stained images.

**Figure 3:**
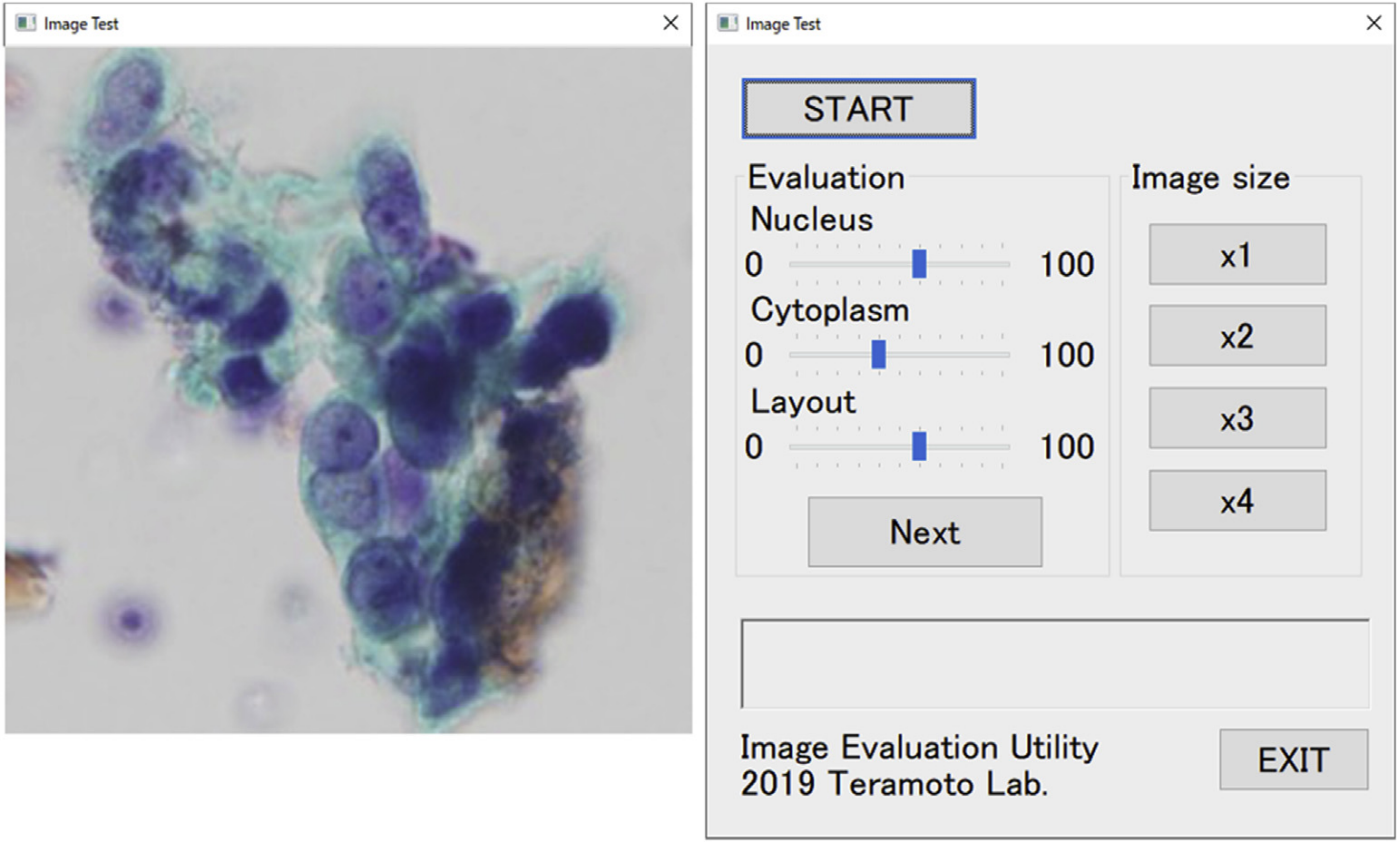
Original software for visual evaluation.

Visual evaluation of image quality was performed by three pathologists (cytology specialists) and three cytotechnologists. Image quality was evaluated from 0 to 100 points for authenticity of cell nucleus (external shape of cellular nucleus, chromatin, nucleolus state), cytoplasm, and cell layout. As shown in Figure 3, we developed and used original evaluation software, which shows real images and converted images at random for visual evaluation. The above evaluations were conducted using Microsoft Surface GO. The display conditions of the PC monitors were standardized and the same ones were used for evaluation.

Moreover, to make a general consideration of the translated image, evaluation comments regarding overall image quality were collected from the observer after the above-mentioned visual evaluation.

## 3 Results

**Figure 4:**
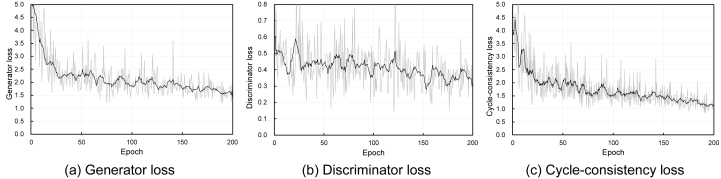
Generator, discriminator, and cycle-consistency loss curves. Gray lines are raw data and black lines are smoothened data.

The losses in CycleGAN include generator loss, discriminator loss, and cycle consistency loss. These curves in the training phase are shown in Figure 4.

**Figure 5:**
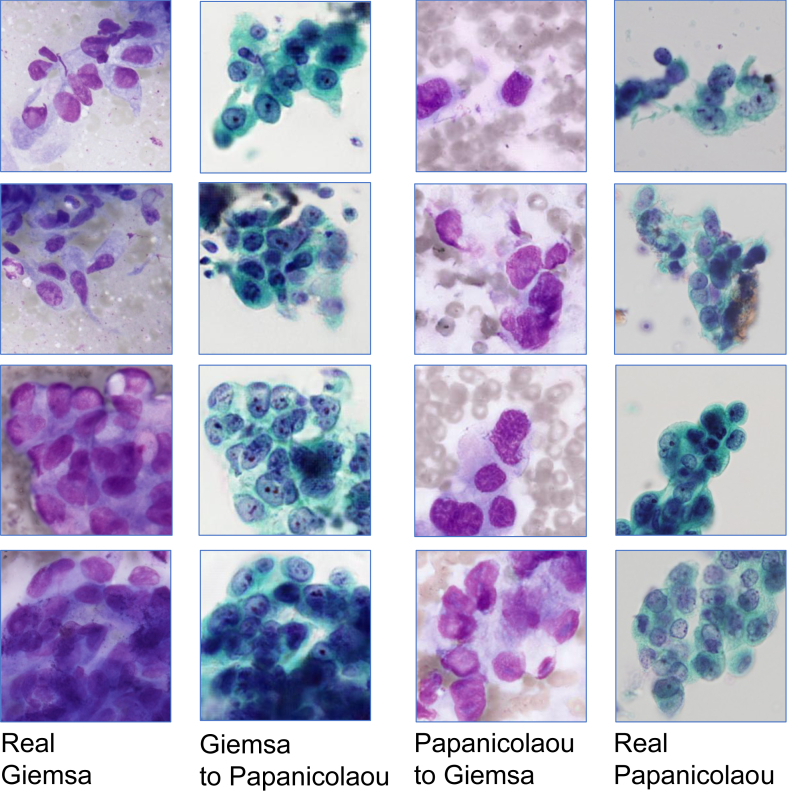
Actual and translated images for adenocarcinoma specimens.

**Figure 6:**
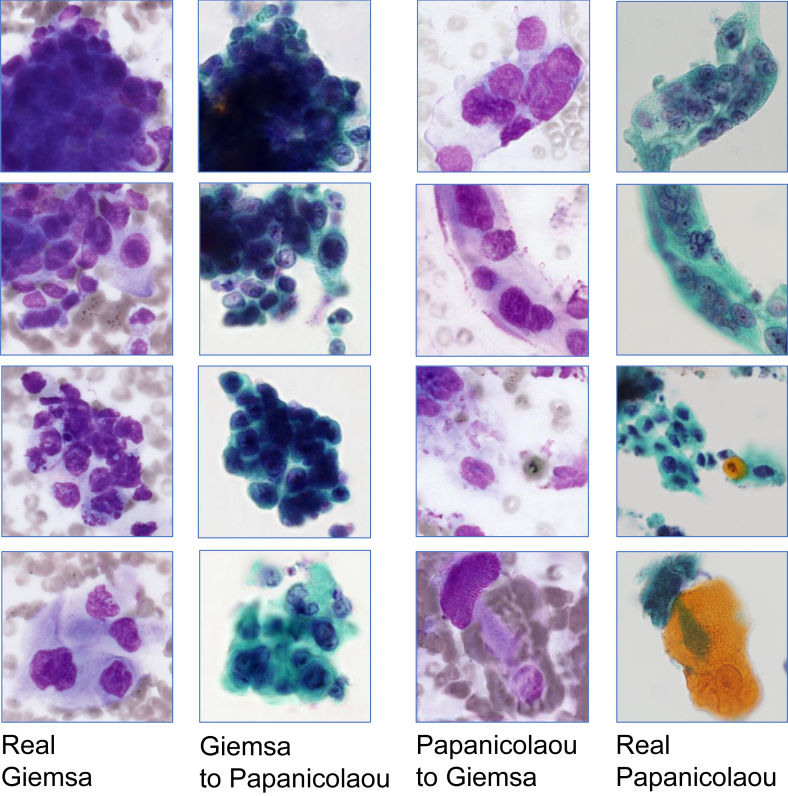
Actual and translated images for squamous cell carcinoma specimens.

**Figure 7:**
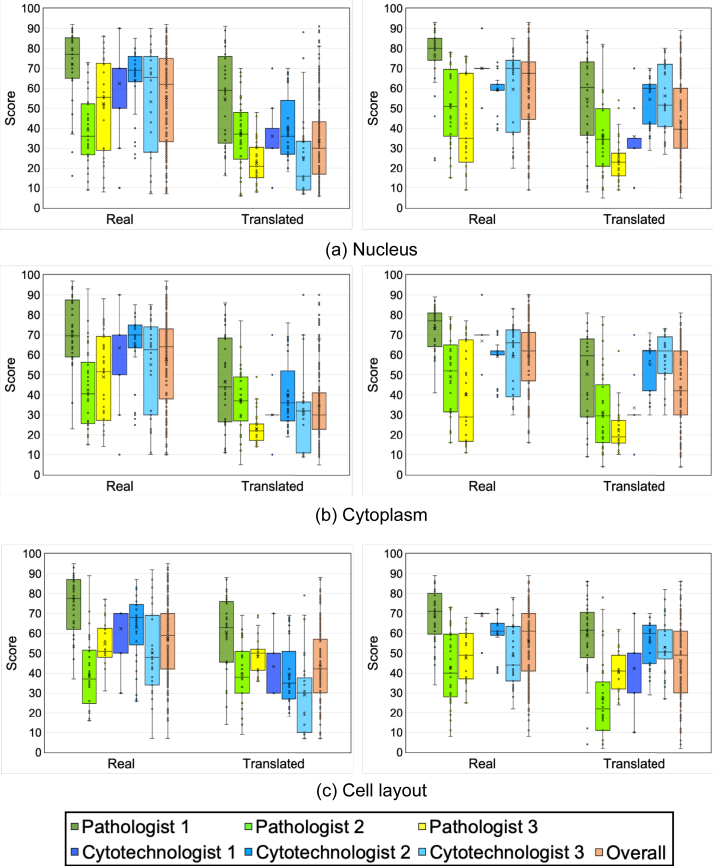
Box plots of visual evaluation results (Left: Giemsa to Papanicolaou, Right: Papanicolaou to Giemsa).

The results of the mutual translation of images from Giemsa-stained and Papanicolaou-stained specimens of adenocarcinoma and squamous cell carcinoma are shown in Figures 5 and 6, respectively. The results of the visual assessment of image quality by six observers are plotted on the box whisker diagram, as shown in Figure 7.

Unpaired Student's *t*-test was also performed on the evaluation results of the image quality of the real and translated images for the six observers, and p-value results were <0.001, confirming that there was a significant-difference in the scores between the real and translated images.

**Figure 8:**
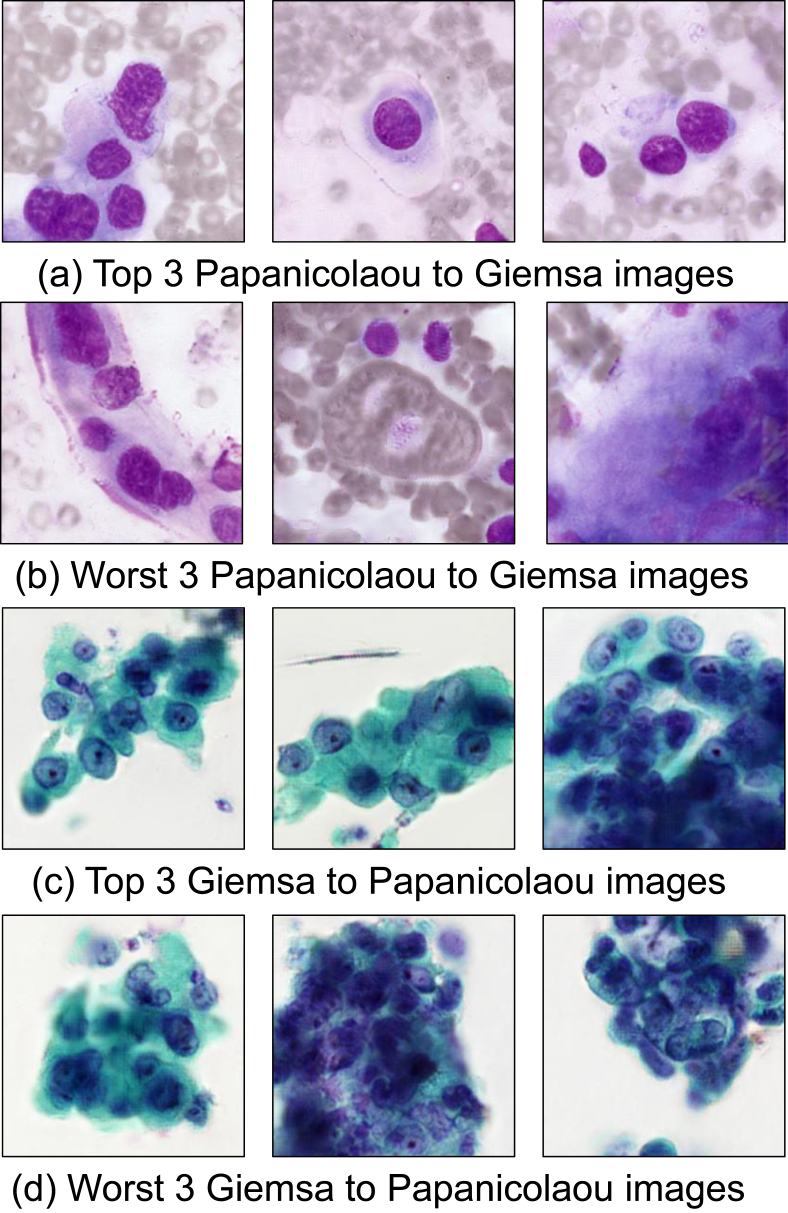
Best-three and worst-three translated images.

Next, the sum of the evaluation results of the three items evaluated by the six observers was calculated for each image, and the best- and worst-three images are shown in Figure 8.

## 4 Discussion

As for Papanicolaou-stained images translated to Giemsa-stained images, faithful representations of blue cell nuclei and light green cytoplasm were obtained. In addition, the erythrocytes were removed, and nucleoli and chromatin that are normally difficult to visualize using Giemsa staining appeared. In many cases, out-of-focus nuclei appeared in the translated Papanicolaou-stained images in the cytoplasmic regions in Giemsa-stained images.

When Papanicolaou-stained images were converted to Giemsa-stained images, the nuclei and cytoplasm were converted to purple, and many red blood cell patterns appeared that were not included in the Papanicolaou-stained images. Giemsa stain tends to render cell nuclei thinner and more spread out because the cells are dried to prepare the specimen. The nuclei in the translated Giemsa-stained images were large, and this feature was reproduced. In addition, in Papanicolaou-stained specimens, there was a mixture of focused and unfocused cells, since specimens maintain the three-dimensional structure of cells. The number of nuclei was reduced by the loss of unfocused nuclei as a result of translating them into Giemsa-stained images with thin structures.

In terms of visual evaluation results, although there was some variation among observers, overall scores for real images were higher than for translated images, with statistically significant differences (P value <0.001) for all evaluation items.

However, there was an overlap between scores for the real and translated groups, which suggested that there was a mix of well- and poorly-transformed images. Therefore, the best three and worst three images with overall scores in the observer test were shown in Figure 8. When we interviewed observers regarding these images, we obtained the following comments. As for the best-three images, the characteristics of nucleus, cytoplasm, and cell layout specific to the tissue type were well-represented. In contrast, the worst-three images shown in Figure 8(c) were out of focus (Figure 8(c) left), the relationship between the cell nucleus and the cytoplasm was broken, the shape of the cell nucleus was disrupted (Figure 8(c) center and right), and artifacts were observed (Figure 8(c) right). In Figure 8(d), keratinized cells (stained orange with Papanicolaou stain) were not correctly converted (Figure 8(b) center), the cytoplasmic margins were unnatural (Figure 8(b) left), and the intracytoplasmic state was unclear (Figure 8(b) right). These might be improved by using more images to train CycleGAN.

The stain translation technique investigated in this paper can complement the specimen in conditions where only single stained specimen is available. It also has potential applications in the massive training of artificial intelligence for cell classification, and education of trainee cytotechnologists and pathologists.

In this study, we introduced a method to convert the staining of lung cytology specimens and examined the possibility of applying this to diagnosis. In cytology, cells collected by biopsy are stained on glass, and different stains cannot be applied to the same cells. Here, we focused on CycleGAN as an image transformation technique using unpaired images. In future studies, we aim to incorporate new models into our research and investigate whether they can produce images that are further improved.

In this method, we employed data augmentation to prevent over fitting and mode collapse. However, the effects of data augmentation are often limited [8]. Future work includes collecting many additional images to improve the image representation capability of the transformed images. In addition, it will be necessary to utilize the deep learning method for cell differentiation and tissue type classification.

## 5 Conclusion

In this study, we developed a mutual conversion technique involving Giemsa- and Papanicolaou-stained images using CycleGAN. Experimental results indicated that proposed stain translation technique may be useful for the diagnosis, artificial intelligence, and education.

## Declarations

### Author contribution statement

A. Teramoto: Conceived and designed the experiments; Performed the experiments; Analyzed and interpreted the data; Wrote the paper.

A. Yamada, K. Imaizumi: Contributed reagents, materials, analysis tools or data; Wrote the paper.

T. Tsukamoto: Conceived and designed the experiments; Performed the experiments; Wrote the paper.

Y. Kiriyama, E. Sakurai, K. Shiogama, A. Michiba: Performed the experiments; Contributed reagents, materials, analysis tools or data.

K. Saito, H. Fujita: Conceived and designed the experiments; Wrote the paper.

### Funding statement

This research was partially supported by a Grant-in Aid for scientific research (No. 20K08060) from the 10.13039/501100001700Ministry of Education, Culture, Sports, Science and Technology, Japan. There was no additional external funding received for this study.

### Data availability statement

Data will be made available on request.

### Declaration of interests statement

The authors declare no conflict of interest.

### Additional information

No additional information is available for this paper.
